# Delayed illness recognition and multiple referrals: a qualitative study exploring care-seeking trajectories contributing to maternal and newborn illnesses and death in southern Tanzania

**DOI:** 10.1186/s12913-019-4019-z

**Published:** 2019-04-11

**Authors:** Donat Shamba, Tara Tancred, Claudia Hanson, Juddy Wachira, Fatuma Manzi

**Affiliations:** 1 Department of Health Systems, Impact Evaluation and Policy, Ifakara Health Institute, Dar es Salaam, Tanzania; 2 Department of Disease Control, London School of Hygiene and Tropical Medicine, London, UK; 3 Department of Public Health Sciences-Global Health, Karolinska Institute, Stockholm, Sweden; 4 School of Medicine/AMPATH, Moi University, Nairobi, Kenya

**Keywords:** Illness recognition, Maternal, Newborn, Care-seeking, qualitative study, Tanzania

## Abstract

**Background:**

Maternal and neonatal mortality remain high in southern Tanzania despite an increasing number of births occurring in health facilities. In search for reasons for the persistently high mortality rates, we explored illness recognition, decision-making and care-seeking for cases of maternal and neonatal illness and death.

**Methods:**

We conducted 48 in-depth interviews (16 participants who experienced maternal illnesses, 16 mothers whose newborns experienced illness, eight mothers whose newborns died, and eight family members of a household with a maternal death), and five focus group discussions with community leaders in two districts of Mtwara region. Thematic analysis was used for interpretation of findings.

**Results:**

Our data indicated relatively timely illness recognition and decision-making for maternal complications. In contrast, families reported difficulties interpreting newborn illnesses. Decisions on care-seeking involved both the mother and her partner or other family members. Delays in care-seeking were therefore also reported in absence of the husband, or at night. Primary-level facilities were first consulted. Most respondents had to consult more than one facility and described difficulties accessing and receiving appropriate care. Definitive treatment for maternal and newborn complications was largely only available in hospitals.

**Conclusions:**

Delays in reaching a facility that can provide appropriate care is influenced by multiple referrals from one facility to another. Referral and care-seeking advice should include direct care-seeking at hospitals in case of severe complications and primary facilities should facilitate prompt referral.

**Electronic supplementary material:**

The online version of this article (10.1186/s12913-019-4019-z) contains supplementary material, which is available to authorized users.

## Background

Despite some progress, maternal and newborn mortality have been slow to decline, particularly in Sub-Saharan Africa. Newborn mortality now represents 46% of all under-five deaths globally, rising from about 38% in 1990 [1, 2]. There is considerable need to strengthen healthcare to reduce maternal and newborn mortality to less than 70 per 100,000 live births and to less than 12 per 1000 live births from 2015 to 2030 respectively, as per the Sustainable Development Goals [3].

In Tanzania, recent studies have reported a significant increase in facility delivery to 63% across the country (2010–2015), and more than 80% in the southern zone (2012–2013) [4, 5]. Despite these marked increases, both maternal and newborn mortality remain persistently high. Furthermore, 94% of women have at least one antenatal visit, however, knowledge about danger signs is reported to be still insufficient [4, 6]. As of 2015, the maternal mortality ratio was estimated to be 398 per 100,000 live births [7], and neonatal mortality rate was 22/1000 live births [8]. These mortality estimates suggest that the quality of healthcare services is inadequate when addressing maternal and newborn care. Health system bottlenecks, including a lack of skilled birth attendants and inadequate medical supplies and equipment, create barriers to reducing maternal and newborn mortality [6, 9, 10]. Traditional practices and poor knowledge of the danger signs during pregnancy and after delivery for both mother and child have been reported to contribute to the delays in illness recognition and care-seeking [11–14].

Identification of potentially life-threatening complications as well as timely and appropriate care-seeking is essential to decrease maternal and neonatal mortality. In 1994, Thaddeus and Maine developed the ‘Three Delays’ model, which organizes barriers to identification and care-seeking for maternal complications into three categories: 1) delays in deciding to seek care; 2) delays in reaching a facility; and 3) delays in receiving quality care [15]. This framework has also been applied to newborn health [16]. The first delay includes both recognition of complications and decision to seek care. The second delay describes delays in reaching care such as transportation barriers. Families need to be supported to understand when, where, and how to access the required care and to act in a timely manner on this knowledge [4, 6, 17]. The third delay includes the delays in the facilities before required care is provided. Here, poor quality of care can present a significant barrier.

Our study was part of a USAID-funded TRAction project that aimed to systematically explore illness-recognition and care-seeking behaviour in the context of community-led health systems interventions to improve maternal and newborn health across seven countries [18]. In Tanzania, this project build on the Expanded Quality Management Using Information Power (EQUIP) project that employed quality improvement at community, health facility and district levels in one intervention (Tandahimba) and one comparison district (Newala) in Mtwara region of Southern Tanzania [19, 20]. In this context, the paradox of high facility delivery alongside high maternal and newborn morbidity and mortality was observed. This paper aims to explore delays in illness recognition and care-seeking using the three delays framework to better understand high maternal and newborn mortality rates within the context of high facility delivery in southern Tanzania.

## Methods

### Study area

The study was conducted in two districts in southern Tanzania (Tandahimba and Newala districts) of Mtwara region in 2015, where the EQUIP study was previously implemented [19, 20]. Most inhabitants of these rural areas belong to the Makonde ethnic group, who primarily engage in subsistence farming. Although most people speak the language of their ethnic group, Kiswahili is widely spoken [21].The uptake of facility births here is high at over 80%, although the neonatal mortality rate is still above 30 per 1000 live births, which is higher than the national average; the national maternal mortality ratio is 556 deaths per 100,000 live births [4, 5].

### Sampling

As we were interested in recruiting participants who had experienced maternal or newborn morbidity and mortality, or family members of a woman who died after giving birth, different methods were employed to recruit respondents. These included using community health workers to help identify cases that were reported to them, snowball sampling methods once participants had been recruited [22], and referrals by community leaders. From these suggestions, we sampled 48 participants purposively [23, 24] from across eight different wards—a sub-district administrative structure—within the two study districts, with differing levels of uptake of facility care. The sample size was chosen to give a sufficient number of interviews to explore different contextual factors that may influence illness recognition and care-seeking in relation to both maternal and newborn illnesses and deaths [18]. Participants in FGDs were village leaders who were purposively selected by the field supervisor based on the inclusion of their wards in the study area.

### Data collection

We conducted 48 group in-depth interviews (IDIs) with family members (16 families who had experienced maternal illness, eight families whose family member had died after giving birth, 16 families whose newborn had experienced illness, and eight families whose newborn had died) and five focus group discussions (FGDs) with community leaders [20].

Semi-structured interview guides were designed and pre-tested. All interviews were conducted in Swahili. A debriefing template was provided to the team by the field supervisor and filled in the same day. At the end of each day of fieldwork, the team held a debriefing session and sent a report to the study coordinator, who provided feedback to the team in the field within 12 h during the first week of data collection and within 48 h for the rest of the study period to improve probing and adaptation of interview guides as required. Please see Additional files 1, 2, 3, 4 and 5 for interview and FGD guides.

### Data analysis

All interviews were audio recorded and transcribed verbatim. After completion of data collection, all debriefing notes and IDI and FGD transcripts were imported into NVivo 9 [25] and analyzed by senior project staff with the data collection team. Analysis began with multiple readings of all data. A codebook was developed and used as a point of comparison and discussion between the team carrying out analysis until a high level of inter-rater agreement about codes could be reached. Thematic analysis was used to group codes together into higher-order sub-themes and overall themes, which we related to the three delays. These themes were reached through group consensus. Representative quotations were selected for each theme and are presented in the results section that follows.

### Ethical considerations

Given the sensitive nature of this data collection, a thorough informed consent process was undertaken with each participant, reiterating to them their right to conclude the interview at any point without consequence. Interviewers paid close attention to any distress that participants were under and were instructed to pause or end the interviews as necessary. Written informed consent was obtained from all participants prior to the start of the IDIs and FGD. All transcripts had identifying information removed, and the confidentiality of all participants was protected from the outset and throughout. Audio files and transcripts were stored in password-protected files on secured computers in locked offices, to which only team members had access.

The study received ethics approvals from the internal review board of Ifakara Health Institute and the National Institute for Medical Research. Permission was also secured from the local authorities in the study area prior to making ethics applications and beginning any data collection.

## Results

### Demographic information of cases

The interviews included eight family members who were speaking about maternal deaths. Among the deceased mothers, five were below 25 years, four were multiparous, and four delivered their most recent baby in a facility. Sixteen families were interviewed regarding serious maternal illness, of them, seven mothers were below 25 years. Most (14 of 16) had two or more children, and 12 had given birth in a facility. From informants speaking about newborn deaths, we learned that four out of eight newborns died before their 15th day of life, two before their tenth day of life, and the other two before their fifth day of life. Seven of these eight babies were born at a health facility. Sixteen families were interviewed regarding serious illness for their newborn babies: seven of the 16 were less than 7 days old at the time of the illness, five of were less than 10 days old and four were less than 15 days old. Thirteen of 16 were born at a facility. We conducted five FGDs each with six community leaders. Community leaders included: ward executive officers, village executive officers and village chairmen from the catchment areas of the study.

We identified themes reported by mothers, their partners or relatives as well as community leaders, which we organized using the three delays framework.

### The first delay: deciding to seek care

#### Illness recognition for mothers and newborns

Most interviewed mothers, husbands or other caretakers were able to report on signs and symptoms of conditions that demanded medical care for either a mother or a newborn. Danger signs were regularly mentioned. Husbands reported that they had been educated about danger signs at health facilities and that they were involved in decision-making around care-seeking together with their wives.

*“If I see signs like crying, stopping sucking breast milk, being wobbly, or convulsions, then I will go to the hospital or the dispensary or health center, because we have been told at the dispensary the danger signs for newborn babies.*” (Husband, newborn death, Tandahimba, IDI)

*“The husband himself made the decision to take his wife to the health center. Due to the condition of his wife, he thought that at the health center they would get tests.”* (Mother in-law, maternal death, Newala, IDI)

#### Delayed care-seeking for sick newborns

Despite often knowing at least some danger signs in newborns, parents were not always able to recognize when the baby was ill enough to need medical attention.

*“I was changing her clothes when I started seeing a rash that had pus, and in the afternoon, the rash increased, and whenever the baby would sleep, the more it increased. That’s when I saw that I decided to go to the dispensary.”* (Mother, newborn illness, Tandahimba, IDI)

*“The baby started to have a fever, but she was still breastfeeding. On Sunday was when the baby stopped taking breast milk. She only took very little breast milk and later stopped completely.”* (Mother, newborn death, Newala, IDI)

Previous experience during childbirth helped family members to recognize illness, when what occurred went beyond what might normally be expected.

*“A pregnant woman cannot give birth without certain complications. Bleeding after giving birth is normal, but it depends on the intensity [of the blood loss]. I am a parent, I have children, I know the delivery process.*” (Mother in-law, Maternal illness, Tandahimba, IDI)

#### Women’s autonomy in decision-making

A common pattern was that, despite mothers realizing the severity of their symptoms, decision-making was delayed by waiting for their husband.

*“After giving birth I was bleeding a lot; I was with my aunty at home; my husband was not at home. My aunty boiled water and massaged me. I wanted to go to the hospital but my husband was not at home, I had to wait for him until he came back.”* (Mother, maternal illness, Newala, IDI)

*“The baby was sick and my husband was not around. I had to wait until he came back from the farm. He came back around 5pm, then I told him about the condition of the baby and he told me that I should go to the hospital.”* (Mother, newborn illness, Tandahimba, IDI)

However, some mothers were able to make decisions alone.

*“It was me. You know, when a man has two wives it is a problem. I was just alone, I didn’t get any advice, I just decided by myself.”* (Mother, newborn death, Newala, IDI)

#### Traditional understanding of illness and care-seeking

Traditional beliefs on what is causing illnesses or symptoms were still reported. However, they were not mentioned as barriers to seeking medical care. While the cause of death was perceived in some cases as God’s will, this did not contradict mothers’ confidence that health providers were doing their best to save lives and that their work was relevant and important.

*“I did not suspect anything when I saw my baby getting very sick; I just saw it as a result of the powers and will of almighty God, whereby every baby comes with her own problems. This thing has not occurred to my other babies, but only to this one, so then I decided to go to the hospital*.” (Mother, newborn illness, Tandahimba, IDI)

*“She was treated well at the hospital, and about the death, it was the work of God himself.”* (Mother of the woman, maternal death, Tandahimba, IDI)

### The second delay: reaching a health facility

#### Transport availability in rural areas

Transport is increasingly available in the villages, but arranging transport in the middle of the night constituted a problem.

*“The problem started at 4 a.m. Since there is a problem getting transport in the village, we said that we should wait until in the morning and then go to the hospital. But she delivered at home and we had a problem removing the placenta, which made her bleed a lot, and she died.”* (Mother of the woman, maternal death, Tandahimba, IDI)

Community leaders explained that much had improved and that there was a common agreement that organizing emergency transport is a community responsibility.

*“In our village, and all the five villages in our ward, we have started the emergency [transport] fund, so when there is an emergency for a family that doesn’t have money for transport to go to the health facility, the village leaders use the village fund for transport.”* (Community leader, Tandahimba, FGD)

#### Multiple referrals through different levels of the health facilities

Mothers and their families found it difficult to navigate through the different health facilities. Often, they first sought care at the dispensary level. However, dispensaries are often ill-equipped to deal with complications. Thus, women and their families were referred to a hospital. This increased travel costs and increased the time spent to reach the required care. While district ambulances exist to facilitate referral from the dispensaries to the district hospital, a chronic lack of fuel, vehicle availability, or other problems hinders the referral system.

*“She started bleeding and we thought, ‘it’s better to take her to the health center’…after reaching there she didn’t get any service. The nurses looked at her and told us that we should go to a big hospital, because she lost a lot of blood and they couldn’t help her…we had motorcycle and the patient was in a very bad condition. We asked for help of a car because the hospital was far away, they tried to get us an ambulance but we were told that the ambulance had no fuel.”* (Mother of the deceased, maternal death, Newala, IDI)

*“I saw the baby curling up and crying. I thought that it’s better I take him to the dispensary and get advice. When we reached there one of the doctors said they don’t have medicine for very young babies and he advised us to find castor nut oil and give that to the baby to drink.”* (Mother, newborn illness, Tandahimba, IDI)

**Fig. 1:**
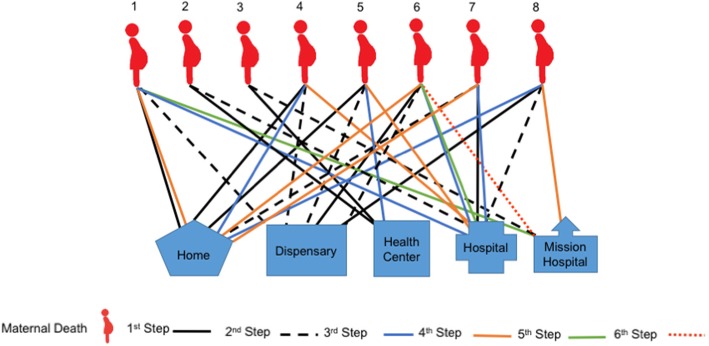
Care-seeking patterns in cases of maternal death

**Fig. 2:**
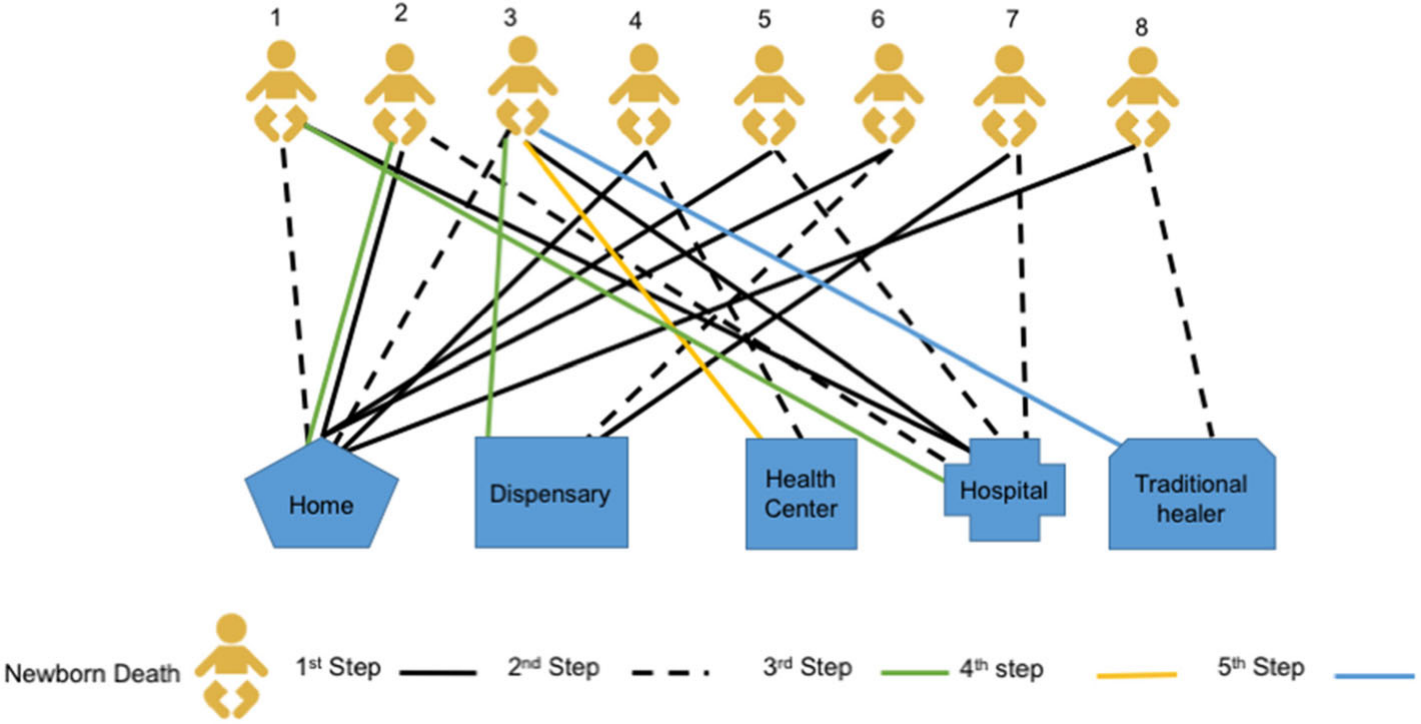
Care-seeking patterns in cases of newborn death

Our data highlight, however, that many women did not simply receive one referral, but were be expected to move between multiple facilities and their homes before reaching a facility in which they could receive care, by which time, it was too late for some. The difficulties navigating the health system and finding the right facility where definitive care could be offered is illustrated in Figs. 1 and 2.

#### Patterns of care-seeking in cases of maternal death

Differences in care-seeking for maternal and newborn death were recorded. For maternal health concerns, most mothers started using home remedies as a first step of care, followed by seeking care at a primary health facility (a dispensary). When the condition worsened, mothers sought care at the tertiary level facility. Among the eight maternal deaths we included in this study five of them moved at least four times, and one woman moved six times (see Fig. 1). Table 1 describes the movement of one of these women.

**Table 1:** An example of care-seeking patterns in cases of maternal death

Case study for woman 1: a mother started bleeding while at home, and her caregiver started to help her with home remedies as the first point of care-seeking. The second point of care was at the dispensary. But she could not get the required service. The third point of care was at the district hospital. The mother still didn’t get the care she needed due lack of medical supplies and money. The family needed to raise enough money to cover medical expenses and living costs. They were discharged and returned home, where the family mobilized financial resources. While at home the patient used home remedies with some medicine from the facility, but the conditioned worsened. The fifth step of care-seeking was to the mission hospital, where the mother died.	
Case study for woman 5: she was eight-months pregnant. She had a high fever, abdominal pain, and was bleeding. Her mother and her husband took her to the dispensary. At the dispensary, the nurse told the family that the facility did not have medicine and equipment to help the patient and referred the mother to the health center. At the health center, the woman was checked by the nurse and the nurse realized that the mother had a stillbirth. At this time, the nurse did not tell the mother or the family of the result of her assessment, instead she referred the patient to the district hospital. On the way, the woman started bleeding and continued to complain about the headache. When they reached at the district hospital, the mother was assessed again and she was told that the baby had died and the blood pressure of the mother was very high. At this point, the mother was unconscious, and the doctor suggested that the patient needed a caesarean section. One day after the surgery, the mother died.	

### The third delay: provision of appropriate care

#### Inadequate care outside of the mission hospitals

As seen in Table 1, most narratives mention the lack of drugs and equipment in facilities as one reasons why care was delayed. In addition, some mentioned that health providers lacked the necessary skills to deal with complications. This was especially a problem in dispensaries, but also at higher level facilities. Difficult cases where often referred to the mission hospital outside the district.

*“When we reached there, we were told that they don’t have the equipment and medicine to help her because she was bleeding severely, and that at that dispensary, they don’t do blood transfusions. The nurse told us that we had to go to another facility so that our patient could get blood and other services*.” (Husband, maternal death, Newala, IDI)

#### Patterns of care-seeking in cases of newborn death

Most care-seeking for newborn illnesses started at the primary health facility (dispensary) level. Families reported dissatisfaction with the care that they received, resulting in moving between multiple health facilities offering different levels of care. Among the newborn deaths we included in this study, most newborns were moved 2–3 times between facilities and homes, and one babies moved five times (see Fig. 2). These movements are illustrated in more detail in Table 2.

*“I decided to report the baby’s problem at the dispensary. The baby was given an injection, but they told me that the baby was too young, so it is difficult to be prescribed with other medicine without a proper diagnosis. Then we decided to go to the mission hospital, but it was too late and the baby died.”* (Mother, newborn death, Newala, IDI)

**Table 2:** An example of care-seeking patterns in cases of newborn death

Case study for newborn 1: The baby had a fever and was taken to the district hospital. At the hospital, the baby was given an antibiotic injection and discharged 2 days after being admitted. At home, the condition of the baby deteriorated, such that he was not able to breastfeed. Her mother decided to give the baby water mixed with sugar. The condition of the baby worsened and her mother decided to go to the hospital. When she arrived at the hospital it was too late, and the baby died immediately after arriving at the hospital.	
Case study for newborn 3: The family first sought care at the district hospital due to the condition of the child. The second point of care was at home because they were told the baby would be okay if they finished a dose of medicine that was prescribed at the facility. The third point of care was at the dispensary, because the baby’s condition did not improve. While at the dispensary, the family was referred to the health center where they were admitted for several days and discharged. The mother returned home and decided to go to the traditional healer, but the baby died.	

Sometime patients were referred from public to mission hospitals.

*“She started this on Friday, it continued Saturday and on Sunday the condition worsened. I told my husband that we should go and see the doctor the next morning because the baby was refusing to breastfeed. We took her to the hospital the next morning, but we did not get medicine. The doctor gave us a card and told us to go to the mission hospital*.” (Mother, newborn death, Newala)

#### Financial barriers to accessing care

Mothers complained about the cost of treatment for maternal and newborn illnesses. They were wondering why they needed to pay despite the Tanzanian policy stating that care in pregnancy, childbirth and for children under 5 years of age is free.

*“When I went [to the hospital], I had the expectation of getting maternal services for free, since we are always told that maternal and child services are provided for free. So, in the process of searching for money to get what we had been prescribed with, we had challenges in getting the money in time…it is hard to get money … So, we had to delay going to the hospital… We waited for the money until the second day…then we went to the hospital.”* (Mother, maternal illness, Tandahimba, IDI)

Community leaders confirmed hearing a lot of complaints in the village on the lack of medicine in most of the health facilities. They also reported that they are saddened to see the challenges take so long to find a solution.

*“The community is saying that the services are not sufficient, most of the time the medicine is not adequate, after one week or after three days, the medicine is out of stock, only one box of medicine has been brought, so when you go there, you will be told that there is no medicine. The doctor is the one who is blamed that he is selling medicine…the amount of medicine that was brought is less.”’* (Community leaders, Newala, FGD)

#### Lack of professionalism in health facilities

Families and mothers also reported that health workers were not always reacting in a professional way. Some health workers refused to provide care for various reasons.

*“I delivered at the health center in a different village then I returned home. I stayed for three days and the baby was not okay. I went to the dispensary that is closer to where I live…to seek for advice. They told me that I was supposed to get advice from facility where I delivered…I told them that after I delivered in that facility, I was asked to go home. But they refused to help me … I came back home where I stayed for two days, then the baby died.”* (Mother, newborn death, Tandahimba, IDI)

*“What [the healthcare worker] did was walk around the room, and then he said that the condition of our patient was not bad, without giving us enough explanation. We were not satisfied because he looked down on us—he didn’t respect us.”* (Mother in-law, maternal illness, Newala, IDI)

Some respondents also reported that they felt that healthcare workers disclose patients’ confidential information. The lack of trust in confidentiality was confirmed by community leaders as a major problem.

*“I was seated with a group of youth one day and they were saying that most doctors break the doctor-patient confidentiality. They were saying that there are some diseases that the doctor needs to keep confidential, but most doctors fail to do that. If this patient comes to realize that everybody around knows that they are sick, they become very embarrassed.”* (Community leaders, Newala, FGD)

## Discussion

Our study indicates that maternal illnesses were recognized in a timely manner while the severity of newborn conditions were often misjudged. Decisions on care-seeking involved both the mother and her partner and primary facilities were first consulted. Delays in care-seeking were reported in absence of the husband, at night, or when the severity of the newborn illnesses was not clear. Mothers and families experienced difficulties navigating the pyramidal health system, often requiring multiple trips between home and different health facilities, and also in preparing money for a hospital stay. Primary facilities were lacking the capacity to stabilize women and newborns and to organize referral to an appropriate hospital.

In Tanzania, birth preparedness and facility delivery is becoming increasingly the norm, even in rural settings [5, 26–28]. While this is progress, our study reveals that barriers to care-seeking remain, which might help to explain the high mortality rates.

Our study indicated some cultural shifts. For example, in our study we describe that decision-making was mostly done in consultation with the husband or partner, while qualitative studies from 9 years ago in the same areas described female relatives as the main decision-maker [11]. Our interviews describe the primary facility, the dispensary, as the main first point to seek care. This is in contrast with other studies from Tanzania and elsewhere where a reliance on traditional medicine is still important [29–31]. This change to greater reliance on formal healthcare corresponds with the changing norm of delivering in facilities described recently in studies from Tanzania [26–28]. A third change we observed was that certain obstacles that were described in the past were not mentioned in our interviews, notably, the fear of wild animals, which hindered families from travelling to receive formal health care services [32]. Several studies from East Africa have described that care-seeking for newborn illness is constrained by customs that do not allow that the newborn to be taken out of home the first days of life [13, 33, 34]. In our study, no interviewees reported such customs as barriers for care-seeking.

### Navigation

Our study described the difficulties of mothers and families navigating through the different levels of the healthcare system. We describe a lengthy pattern of care-seeking for maternal and newborn complications—from a primary facility to a tertiary care facility when care was not available, service was dissatisfactory, or when complications persisted—then coming back home to reorganize resources to return to a public hospital or to seek care from a mission facility. This observation raises the question of the role of the primary facility for maternal and newborn illnesses. Many studies from southern Tanzania and other parts of the country have described that dispensaries are not equipped to provide emergency obstetric care [6, 10, 28, 35, 36]. However, as dispensaries are close to higher-level facilities, they could play an important role in helping families by assisting them to choose the right facility and organizing effective referral [37]. Our study reveals that the role of primary facilities for the management of maternal and newborn complications is limited.

Community complaints regarding quality of care included the lack of drugs and the subsequent need to purchase missing drugs and equipment in private pharmacies. Participants were aware that maternal and newborn health services should be offered for free as it is a national policy and they expressed dissatisfaction with this common practice. The lack of confidentiality and unprofessional treatment even including refusals to provide care were other themes. Unprofessional behaviour and disclosure of confidential information have been also reported by other studies from Tanzania [38, 39]. These issues further limit the confidence in lower-level health facilities, and perhaps contribute to care-seeking elsewhere.

### Implications for policy

The findings here suggest, as has been called for elsewhere [6, 10, 28, 35, 36], that there is a need to strengthen the quality of maternal and newborn care at primary health facilities. In particular, capacitating health facilities at lower levels to recognize potential problems during labour and in antenatal and postnatal care, to stabilize women, and to effectively refer them to more capable facilities. These actions would potentially prevent unnecessary morbidity and mortality. These changes have been advocated for within the National Road Map Strategic Plan to Improve Reproductive, Maternal, Newborn, Child and Adolescent Health in Tanzania (2016–2020) [40]. Specifically, strengthening of care for mothers and newborns across the continuum, including emergency obstetric care, through training of staff. Further, improving referrals to and strengthening the quality of care at higher-level facilities capable of providing comprehensive emergency obstetric care would be necessary to ensure timely, appropriate, life-saving care. Improvements to referral have also been advocated by the Tanzanian government, beginning with procurement of more ambulances, community sensitization, initiating voucher schemes to cover transport costs, and improving communication between facilities. Continued impetus for these changes is supported by studies like ours, which highlight the ongoing existence of gaps.

### Implications for future research

To align with the national government’s goals of strengthening referral capacity, it would be important for further studies to identify the most effective ways of doing so. Further, understanding different modalities of community engagement to achieve this goal would be of value. Understanding the rationale behind care-seeking—as we have demonstrated here—would be of enormous benefit, as this may inform community education and mobilization around referral.

### Study limitations

The families provided narratives of events that had occurred some time ago (up to a six-month recall). It is likely that they might have forgotten some of the key aspects of the events and steps in decision-making and care-seeking. However, given that these were major events, the descriptions provided are likely to be more accurate than for recall of less significant events. Also, we do not have clinical data to verify the nature of health problems faced by the various cases. We depended entirely on the descriptions by families. Social desirability bias is likely—some families may have reported that they intended to seek care in facilities, as they felt that this was expected. However, there was considerable agreement around this point among all study participants, including in FGDs with community leaders.

## Conclusions

While illness recognition was relatively good for maternal illnesses, mothers and families faced difficulties interpreting the severity of newborn illnesses. Interventions to support early recognition and care-seeking for newborn illnesses at the community level need to address these challenges. They might include strategies such as allowing consultation with health workers over the phone, community education around danger signs, and community sensitization around the need for timely decision-making.

Many families faced substantial delays receiving needed care because of consulting several health facilities before finally being taken care of. Thus, the second delay from the decision for care-seeking to reaching a facility that can provide definitive care was determined here by multiple referrals from one facility to another. Policy-makers need to review the role of primary facilities and the functioning of referral systems to enable timely referral to a facility adequately equipped to deal with complications. Community approaches to improve illness recognition and care-seeking should include informing the community about the limitations of primary facilities in addressing severe complications to reduce frustration resulting from multiple referrals. Referral advice for severe complications should be to go directly to a hospital.

## Additional files

Additional file 1: Interview guide – event narrative maternal illness. (DOC 85 kb)

Additional file 2: Interview guide – event narrative maternal death. (DOC 84 kb)

Additional file 3: Interview guide – event narrative newborn illness. (DOCX 44 kb)

Additional file 4: Interview guide – event narrative newborn death. (DOCX 42 kb)

Additional file 5: FGD guide – community leaders. (DOC 68 kb)

## Abbreviations

FGDs: Focus group discussions
IDI: In-depth interview
USAID: United States Agency for International Developments
