# Supplementary material for: Delayed illness recognition and multiple referrals: a qualitative study exploring care-seeking trajectories contributing to maternal and newborn illnesses and death in southern Tanzania
Source: BMC Health Serv Res. 2019 Apr 11;19:225. doi: 10.1186/s12913-019-4019-z (PMC6460539; doi:10.1186/s12913-019-4019-z)
Supplement: Supplementary file 1 — Interview guide – event narrative maternal illness. (DOC 85 kb) [file 12913_2019_4019_MOESM1_ESM.doc]

**Event Narrative – Women who reported excessive bleeding after birth**

Focal woman ID **___ ___ ___ ___ ___** Interviewer ID **___ ___ ___ ___** Note Taker ID **___ ___ ___ ___**

Interview date: **__ __ / __ __ / ___ ___** Illness event date: **__ __ / __ __ / ___ ___**

**(***DD/MM/YY***) (***DD/MM/YY***)**

**INTERVIEWER – VERIFY THAT ILLNESS OCCURRED WITHIN 6 MONTHS OF INTERVIEW DATE**

| **Participant** | **Present during Interview?** | **Present during Illness Event?** | **Main caregiver? [MARK ONLY ONE]** |
| --- | --- | --- | --- |
| Focal woman (woman with excessive bleeding) |  |  |  |
| Focal woman’s husband or partner |  |  |  |
| Focal woman’s mother |  |  |  |
| Focal woman’s mother-in-law |  |  |  |
| Focal woman’s sister |  |  |  |
| Focal woman’s sister-in-law |  |  |  |
| Birth attendant (define type): ____________ |  |  |  |
| Community health worker |  |  |  |
| Focal woman’s neighbor or friend |  |  |  |
| Focal woman’s daughter |  |  |  |
| Focal woman’s daughter-in-law |  |  |  |
| Focal woman’s other relative (define): ______________ |  |  |  |
| Focal woman’s other (define): ______________ |  |  |  |

Time interview started: **:**

***Ask the group to take a moment to think back to the birth and their experience with a lot of bleeding after childbirth. Tell the group that we will begin with the woman’s memory of the event and then invite the others to contribute to the narrative from their own memories. Since people often see things differently, it is ok if there are differing pieces to contribute to the overall story of what happened.***

***First, ask the woman (focal woman) to begin by telling her story of what happened. Remind her that you are interested in everything that she can remember from the time of birth to the point at which the bleeding slowed down or stopped.***

**Questions:**

**Part A: Recognition**

1. Please describe the types of problems that you experienced.

***From the initial open-ended question, make sure to ask the following questions for each type of problem or symptom they mention:***

1. When did this start?
2. What was it that made you think something was wrong?
   1. Probe for descriptions of what was seen or felt (symptoms). Example: What were you feeling at the time?
3. How serious did you think the problem was?
   1. Probe on reasons on why they thought (or didn’t think) the problem was serious.
      1. Example probes: What made you think that?
      2. Why? Please explain.

| Interviewer Checklist | | |  |
| --- | --- | --- | --- |
| Did you get information on: | Yes | No |  |
| What was noticed? (all the signs and symptoms) |  |  |  |
| When it started? |  |  |  |
| What made them think it was serious? |  |  |  |
| If you answered no to any question, make sure to go back and probe further on those areas. | | | |

**Part B: Care Seeking**

1. What did you and your family do **first** to address this problem?
   1. Who (what kind of provider) provided care and where (what place) was treatment or care sought, if any? **(PROBE FOR CARE AND TREATMENTS INSIDE AND OUTSIDE OF THE HOME)**
      1. Probe on type of provider and/or place of care (such as level of facility or informal sector).
      2. If care was inside the home, probe on:
         1. Whether treatment/care came from outside? If so, from whom and where?
         2. How was treatment/care given? By whom and how often?
   2. Who was involved in decision-making around what to do, if anyone?
      1. Probe on getting information on all the actors involved and if there were conflicting opinions.
   3. What issues or things were considered in the discussion about seeking care/treatment from this provider or place?
      1. Probe on factors to seek care. If care was in the home, probe on factors that prevented going out.
   4. Who made the final decisions?
      1. Probe on whether they agreed with these decisions.
   5. What factors were considered in determining the types of treatment or care needed from this provider or place?
   6. How long did it take to make the decision to seek this type of treatment or care?
      1. Probe on time from recognition to action.
   7. How did you and/or your family feel about the care/treatment received? Probe on reasons for satisfaction or dissatisfaction.
2. What did you and your family do **next** to address this problem?

REPEAT QUESTIONS 5a-g FOR EACH STEP OF CARE. **[CONTINUE ASKING THESE QUESTIONS UNTIL LAST TREATMENT/CARE WAS SOUGHT]**

| Interviewer Checklist | | |  |
| --- | --- | --- | --- |
| Did you get information on: | Yes | No |  |
| The sequence of events for seeking care and/or treatment both inside and outside the home? |  |  |  |
| The type of provider providing care? |  |  |  |
| Location of care and/or place of referral? |  |  |  |
| Factors considered in making the decision? |  |  |  |
| Who was involved in the decision-making process? |  |  |  |
| The time it took from recognition to deciding on treatment/care and then receiving it? |  |  |  |
| How and why care was sought for each step? |  |  |  |
| If you answered no to any question, make sure to go back and probe further on those areas.  Make sure you have a **narrative** and not just a list of events and places. We want a detailed description of what happened and why it happened. | | | |

1. What happened in the end? How is your health now? How is your baby’s health?
2. Invite each of the participants to add to the woman’s narrative from their own memories of what happened.
3. Ask the group to discuss any differences in their memories of the event among themselves.
4. Record the group’s responses on the Postpartum Hemorrhage Event Timeline.
5. When they appear to have finished, review and verify the information you have recorded with the participants. Ask them if there is anything you have misunderstood or that they would like to add.

**Part C: General group questions on causes and lessons learned**

***Now I’d like to ask the group some general questions concerning this experience.***

1. Why does the group think this problem with a lot of bleeding after childbirth happened?
   1. Probe on any reasons given and possible past experiences.
2. How has this experience influenced you?
3. Is there anything else you would like to share with me that we haven’t discussed?

***Finally, I’d like to ask you a few additional questions about your last pregnancy:***

1. How old were you when you had your last pregnancy?

____ ____ years

1. Where did the delivery take place?

At home…………………1

On the way to a health facility…………….2

Hospital ……………3

Other health facility………….4

Other…………..5

1. How was the baby born?

Vaginally, spontaneous……………….1

Vaginally, with forceps, vacuum, or other instrument……………………..2

Caesarean section……………………3

1. How many pregnancies (including the last pregnancy) have you had?
2. How many antenatal care (ANC) visits did you receive during your last pregnancy? _______

Time interview ended: **:**

***Thank the participants for their time. Remind them that the information will be kept confidential.***

**POSTPARTUM HEMORRHAGE EVENT TIMELINE**

Diagram the event timeline developed by the group in the space provided below, starting at the time of birth and completing with the bleeding slowed or stopped. On top of the line, record key symptoms and the progression of events. Below the line, include actions taken, including seeking treatment/care.

**Birth Resolution**

**Approximate duration of total illness: _____ hours**
