# Supplementary material for: Delayed illness recognition and multiple referrals: a qualitative study exploring care-seeking trajectories contributing to maternal and newborn illnesses and death in southern Tanzania
Source: BMC Health Serv Res. 2019 Apr 11;19:225. doi: 10.1186/s12913-019-4019-z (PMC6460539; doi:10.1186/s12913-019-4019-z)
Supplement: Supplementary file 5 — FGD guide – community leaders. (DOC 68 kb) [file 12913_2019_4019_MOESM5_ESM.doc]

**FGD Guide with village/community leaders**

Informant ID **___ ___ ___ ___ ___** Interviewer ID **___ ___ ___ ___** Note Taker ID **___ ___ ___ ___**

Interview date: **__ __ / __ __ / ___ ___**

***(****DD/MM/YY****)***

| **Name (first only)** | **Age** | **No. of children** | **Ethnicity** | **Occupation/main activity** | **Education** | **Code** |
| --- | --- | --- | --- | --- | --- | --- |
|  |  |  |  |  |  |  |
|  |  |  |  |  |  |  |
|  |  |  |  |  |  |  |
|  |  |  |  |  |  |  |

Time interview started:: **:**

**Section 1: Background information**

1. What is your title?

- How long have you been in this position?

2. What is your role in the community?

- How did you get this role?

**Section 2: Health issues and factors that influence care seeking**

3. What are the main health issues in your community around pregnancy, childbirth, and newborns?

- Probe on why they think these are important issues.
- Probe on how they notice something is wrong in pregnancy, childbirth or in a newborn.

4. If health problems/illnesses arise, what do families typically do?

5. Where do families in your community think is the best place to receive treatment?

Probe on whether families:

- Bring treatment to the home
- Receive home visits from health providers
- Go to a public health facility
- Go to a private health facility
- Seek treatment with informal sector providers within the community (e.g., traditional healers, spiritual/religious healers, herbalists)
- Try to find if there are differences in where care is ideally provided:
  - antenatal vs labor vs postnatal
  - based on who is sick or having complications (women vs. newborns)

6. Who typically makes decisions around what type of treatment/care to seek?

- Who are the opinion leaders in the community?
- What members of a household usually make decisions to seek care and the type of care to seek?
  - Probe for details on the different roles of decision-makers.

7. Who is a reliable source of information about complications in pregnancy and childbirth in your community? For newborn problems or illnesses?

8. In your opinion, what are the key factors that help families receive the care they need in these situations? What are the key factors that make it difficult to receive the care they need?

- Probe for factors at household, community and health facility level

9. Are there any projects going on in your community to try to help overcome some of these barriers to care? If not, can you think of ways that would help increase access to care?

- Probe about the existence of community savings funds, community transport schemes for emergencies, women’s groups, other schemes to increase access to care.
- What are the challenges to addressing these factors?

**Section 3: Health services available:**

10. What options are available for seeking care in your community?

11. Where do people generally go for routine care for pregnant women, delivery and newborns?

Probe on care for pregnancy, childbirth, and the postnatal/early newborn period, including care provider and place of care.

12. Where do most people in your community seek care when pregnant women, new mothers, or newborns are sick?

Probe further for each below:

- For serious complications for the mother?
- For serious complications for the newborn?
- Probe about both public and private facilities (formal health system) and the informal sector (traditional healers, religious/spiritual healers, herbalists).

13. What is the general opinion in the community regarding the quality of care available at the closest public health facility?

Probe on:

- How do people think about the care available there?
- What are people’s major concerns, if any, with the care there?
- Do people think that better care is available elsewhere? If so, where?
- Do some groups in the community feel more or less positively about the care at the nearest public health facility? Probe about differences between these groups’ regard for the health facility.

14. What is the overall opinion of the health services available in this community?

- Probe to compare the perceived quality, cost, and accessibility of public vs private facility-based care
- Probe about the perceived quality, cost, and accessibility of informal sector care (traditional healers, spiritual healers, herbalists, etc)

15. Is there anything else you would like to tell me about the health services in your community?

Time interview ended:: **:**

***Thank the participant for his time. Remind him that the information will be kept confidential.***
